# Academy of Sciences, Paris—July

**Published:** 1844-10-19

**Authors:** 


### ACADEMY OF SCIENCES, PARIS—JULY.

#### PSEUDO-MEMBRANOUS INFLAMMATION OF THE BLADDER PRODUCED BY BLISTERS.

M. Morel Lavallèe stated that although, generally speaking, cantharides applied to the skin, exercise no influence over the bladder, they sometimes develope in that organ, owing to individual peculiarity, an inflammation similar to that produced on the skin, and accompanied by the formation of false membranes. The size of the blister appears to have a considerable influence over the occurrence of these accidents. In three cases which M. Morel Lavallée gave, the blisters were very large. One had been applied near the bladder on the hypogastric region; the others had been applied at a considerable distance on the head and the chest. The false membranes are sometimes small, thin, with an irregular festooned margin, whilst sometimes they are as large as half a playing card. In the first instance, they are of a dull-white colour on the adherent one. In one case in which M. Vidal de Cassis was able to examine the bladder after death, its internal surface was red and swollen, like the conjunctiva in blennorrhagic ophthalmia. The symptoms are those of ordinary cystitus. It is worthy of remark that in the cases observed by M. Morel Lavallée, the blister had been powdered with camphor. In the treatment of these cases M. Morel advises vesical injections of emollient fluids, along with poultices, refreshing drinks, &c., at the same time he takes off the blister.—*Lon. Med. Times.*

